# Supplementary figures and images for: Outcomes of stereotactic body radiotherapy for unresectable cholangiocarcinoma: a meta-analysis and systematic review
Source: PeerJ. 2025 Aug 28;13:e19909. doi: 10.7717/peerj.19909 (PMC12399086; doi:10.7717/peerj.19909)

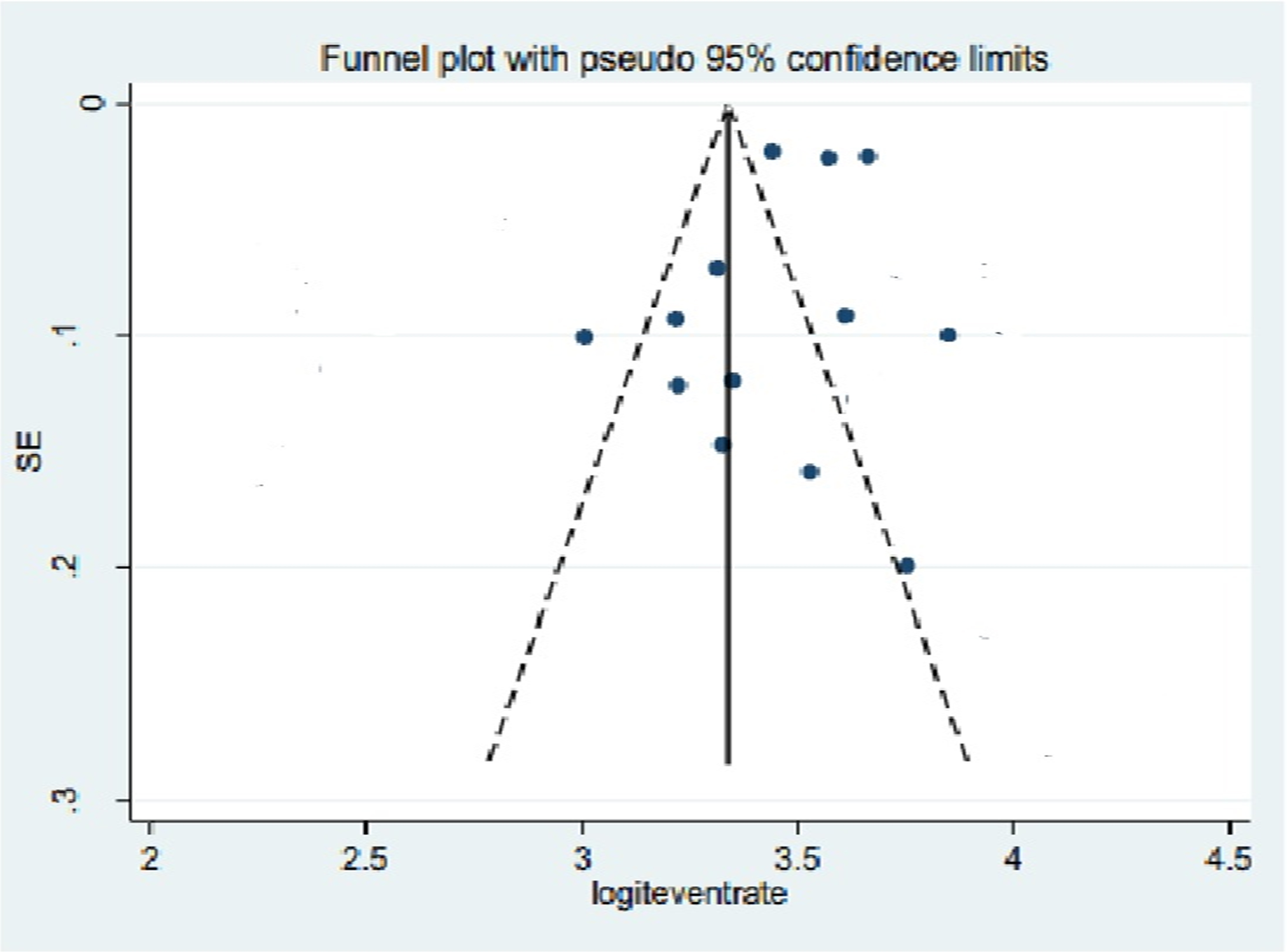

Supplement: Supplemental Information 2 [file peerj-13-19909-s002.jpg]
