# Supplementary material for: Outcomes of stereotactic body radiotherapy for unresectable cholangiocarcinoma: a meta-analysis and systematic review
Source: PeerJ. 2025 Aug 28;13:e19909. doi: 10.7717/peerj.19909 (PMC12399086; doi:10.7717/peerj.19909)
Supplement: Supplemental Information 3 [file peerj-13-19909-s003.docx]

**Supplementary Table 1** The quality evaluations of all included articles according to Newcastle Ottawa Scale

| **Author** | **Year** |  | **Selection** | | | |  | **Comparability** | |  | **Outcome** | | |  | **Total score** |
| --- | --- | --- | --- | --- | --- | --- | --- | --- | --- | --- | --- | --- | --- | --- | --- |
|  |  |  | **(1)** | **(2)** | **(3)** | **(4)** |  | **(1)** | **(2)** |  | **(1)** | **(2)** | **(3)** |  |  |
| Zhang et al. | 2022 |  | **🟑** | - | **🟑** | **🟑** |  | - | - |  | **🟑** | **🟑** | **🟑** |  | 6 |
| Liu et al. | 2017 |  | **🟑** | - | **🟑** | **🟑** |  | - | - |  | **🟑** | **🟑** | **🟑** |  | 6 |
| Thuehoj et al. | 2022 |  | - | - | **🟑** | **🟑** |  | - | - |  | **🟑** | **🟑** | **🟑** |  | 5 |
| Tse et al. | 2008 |  | - | - | **🟑** | **🟑** |  | - | - |  | **🟑** | **🟑** | **🟑** |  | 5 |
| Mahadevan et al. | 2015 |  | **🟑** | - | **🟑** | **🟑** |  | - | - |  | **🟑** | **🟑** | **🟑** |  | 6 |
| Brunner et al. | 2019 |  | **🟑** | - | **🟑** | **🟑** |  | - | - |  | **🟑** | **🟑** | **🟑** |  | 6 |
| Kozak et al. | 2020 |  | **🟑** | - | **🟑** | **🟑** |  | - | - |  | **🟑** | **🟑** | **🟑** |  | 6 |
| Ibarra et al. | 2012 |  | **🟑** | - | **🟑** | **🟑** |  | - | - |  | **🟑** | **🟑** | **🟑** |  | 6 |
| Sandler et al. | 2016 |  | **🟑** | - | **🟑** | **🟑** |  | - | - |  | **🟑** | **🟑** | **🟑** |  | 6 |
| Kopek et al. | 2010 |  | **🟑** | - | **🟑** | **🟑** |  | - | - |  | **🟑** | **🟑** | **🟑** |  | 6 |
| Polistina et al. | 2011 |  | **🟑** | - | **🟑** | **🟑** |  | - | - |  | **🟑** | **🟑** | **🟑** |  | 6 |
| Shen et al. | 2017 |  | **🟑** | - | **🟑** | **🟑** |  | - | - |  | **🟑** | **🟑** | **🟑** |  | 6 |
| Welling et al. | 2014 |  | **🟑** | - | **🟑** | **🟑** |  | - | - |  | **🟑** | **🟑** | **🟑** |  | 6 |

**Selection 0-4🟑**

(1) Representativeness of the exposed cohort

(2) Selection of the non exposed cohort

(3) Ascertainment of exposure

(4) Demonstration that outcome of interest was not present at start of study

**Comparability 0-2🟑**

Comparability of cohorts on the basis of the design or analysis

(1) Study controls for _____________ (select the most important factor)

(2) Study controls for any additional factor (This criteria could be modified to indicate specific control for a second important factor.)

**Outcome 0-3🟑**

(1) Assessment of outcome

(2) Was follow-up long enough for outcomes to occur

(3) Adequacy of follow up of cohorts
